# Computer-aided diagnosis of isocitrate dehydrogenase genotypes in glioblastomas from radiomic patterns

**DOI:** 10.1097/MD.0000000000019123

**Published:** 2020-02-21

**Authors:** Chung-Ming Lo, Rui-Cian Weng, Sho-Jen Cheng, Hung-Jung Wang, Kevin Li-Chun Hsieh

**Affiliations:** a Graduate Institute of Biomedical Informatics, College of Medical Science and Technology, Taipei Medical University; b Graduate Institute of Library, Information and Archival Studies, National Chengchi University; c Taiwan Instrument Research Institute, National Applied Research Laboratories; d Graduate Institute of Biomedical Electronics and Bioinformatics, National Taiwan University; e Department of Medical Imaging, Taipei Medical University Hospital; f Department of Radiology, School of Medicine, College of Medicine, Taipei Medical University, Taipei, Taiwan.

**Keywords:** isocitrate dehydrogenase, glioblastoma, computer-aided diagnosis, ranklet transformation, magnetic resonance imaging

## Abstract

World Health Organization tumor classifications of the central nervous system differentiate glioblastoma multiforme (GBM) into wild-type (WT) and mutant isocitrate dehydrogenase (*IDH*) genotypes. This study proposes a noninvasive computer-aided diagnosis to interpret the status of *IDH* in glioblastomas from transformed magnetic resonance imaging patterns. The collected image database was composed of 32 WT and 7 mutant *IDH* cases. For each image, a ranklet transformation which changed the original pixel values into relative coefficients was 1st applied to reduce the effects of different scanning parameters and machines on the underlying patterns. Extracting various textural features from the transformed ranklet images and combining them in a logistic regression classifier allowed an *IDH* prediction. We achieved an accuracy of 90%, a sensitivity of 57%, and a specificity of 97%. Four of the selected textural features in the classifier (homogeneity, difference entropy, information measure of correlation, and inverse difference normalized) were significant (*P* < .05), and the other 2 were close to being significant (*P* = .06). The proposed computer-aided diagnosis system based on radiomic textural features from ranklet-transformed images using relative rankings of pixel values as intensity-invariant coefficients is a promising noninvasive solution to provide recommendations about the *IDH* status in GBM across different healthcare institutions.

## 1 Introduction

Glioblastoma multiforme (GBM) is the most common and most aggressive glioma in brain.^[1,2]^ Nearly 90% of GBMs are classified as primary, with the remaining 10% being secondary. The prognosis of primary GBMs is grim despite advances in different therapies.^[3]^ Recent genomic characterization of both low- and high-grade gliomas showed frequent mutations in the isocitrate dehydrogenase 1 (*IDH1*) gene and its homolog, *IDH2*.^[4,5]^ These mutations impair *IDH*'s function, and result in accumulation of an oncogenic metabolite, d-2-hydroxyglutarate (d-2HG), within the tumor.^[5,6]^ This metabolite induces epigenetic changes that result in abnormal regulation of gene expressions and cellular differentiation, along with increased levels of hypoxia-inducible factor-1α, which are all important elements of tumorigenesis.^[7–9]^ In the latest World Health Organization tumor classification of the central nervous system, GBMs are classified as wild-type (WT) *IDH* and mutant *IDH* GBMs.^[3]^ The mutant *IDH* GBMs are associated with better prognosis compared to their WT counterparts. *IDH* mutations were associated with prolonged progression-free survival and a trend for prolonged overall survival.^[10]^

Currently, the most commonly applied method to detect *IDH* mutations in GBMs is an immunohistochemical analysis, in which a specific monoclonal antibody that recognizes the R132H amino acid mutation is applied. However, there are still diagnostic challenges because of the partial sampling of lesions and heterogeneity of tumors. Cryan et al also demonstrated a limitation of traditional *IDH1* antibody testing in terms of the sensitivity of the applied antibody.^[11]^ Moreover, it was proven that survival benefits associated with surgical strategies differ based on the *IDH1* genotype in malignant astrocytomas.^[12]^ Therefore, a noninvasive method for preoperative prediction of the *IDH* genotype is important for surgical planning and research in understanding the biology of gliomas.

Magnetic resonance (MR) imaging (MRI) is an ideal solution for characterizing physiologic and molecular features of GBMs in a noninvasive manner.^[13,14]^ MRI equipped with specialized MR spectroscopic techniques was proved to be able to detect the in vivo accumulation of d-2HG, the oncometabolite produced from *IDH* mutations.^[15,16]^ Other MRI techniques, including perfusion and diffusion imaging, were also proposed to distinguish differences between WT and mutant *IDH* GBMs.^[17]^ Interpretation of the *IDH* status from MRIs can be realized from heterogeneous patterns within the tumor area. With the development of textural analyses, pixel-wise correlations present tiny details between tissues which might not be readily recognized by human beings. Additionally, the quantification process has strengthened the clinical utility of MRI.

To provide a more-fitting interpretation of tissue compositions, the quantified texture extracted from the tumor area can be combined in an artificial intelligence classifier to achieve a computer-aided diagnosis (CAD) system.^[18,19]^ However, Buch et al proposed that a lack of standardized scanning protocols for images collected from different institutes may lead to variations in textural analytical features irrespective of the internal architecture.^[20]^ To reduce the effects of such confounding factors, this study proposed a specific CAD model based on ranklet transformation to interpret the *IDH* status through a sophisticated integration of numerous textural features. The ranklet transformation uses relative rankings of pixel values in a local area as intensity-invariant coefficients to emphasize the underlying image pattern. The resulting estimate of the likelihood of there being an *IDH* mutation facilitates the clinical diagnosis in a more-reliable way.

## 2 Materials and methods

### 2.1 The cancer genome atlas and the cancer imaging archive

The data set used in the experiment was from the cancer imaging archive (TCIA; http://cancerimagingarchive.net/*)* established by the National Cancer Institute. Patients who underwent MRI examinations also have *IDH* mutation information in the cancer genome atlas (TCGA). GBM cases were composed of 32 WT *IDH* and 7 mutant *IDH* forms. Materials provided by TCIA and TCGA were used in compliance with all applicable laws, regulations, and policies based on Washington University School of Medicine IRB Protocol 201108194. The necessary approvals, authorizations, participant assurances, informed consent documents, and institutional review board approvals in every institution related to this research were acquired.^[21]^

The MRIs of 32 WT *IDH* GBMs were obtained from Case Western and Henry Ford Hospitals. Seven mutant *IDH GBMs* were collected from Emory University, Henry Ford Hospital, and Fondazione IRCCS Instituto Neuroligico C. Besta. These cases were determined after exploring 291 GBM cases in TCIA where only 15 (5.15%) were found to be mutant *IDH* GBMs. Among these 15, only 7 cases with preoperative contrast-enhanced T1-weighted images (T1WIs) were enrolled in the experiment. *IDH1 R132G* mutation was detected in one case, and the remaining cases had the *IDH1 R132H* mutation. No *IDH2* mutation case can be found in TCIA. The WT *IDH* cases were obtained from 2 of the 4 institutes through consecutive selection in TCGA archive. Due to the insufficient image quality, 8 of 40 WT *IDH* cases were excluded. Patient and tumor characteristics are listed in Table 1.

**Table 1:**
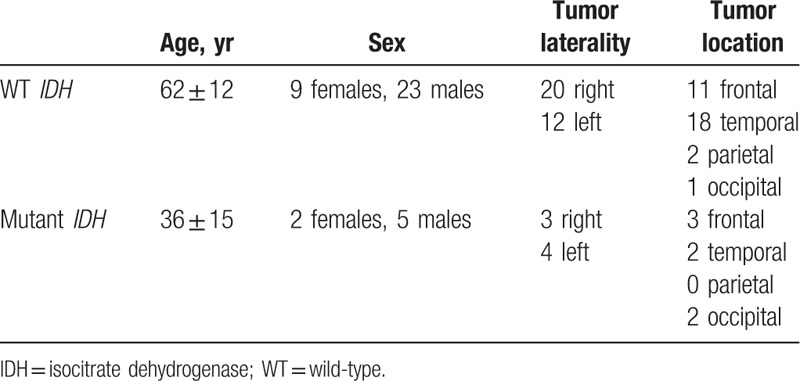
Demographic information of the cohort.

### 2.2 Traditional interpretation by neuroradiologists

Since traditional radiographic features on MRI have been proposed based on univariate analyses that IDH1 mutant tumors were more frequently located at the frontal lobe adjacent to the subventricular zone.^[22,23]^*IDH1* mutant GBMs more likely exhibit a unilateral growth pattern, sharp tumor margins, a lower volume of enhancement, and a homogeneous signal intensity.^[24]^ Three neuroradiologists (KH, with 14 years of experience, HJW, with 17 years of experience, and SJC, with 25 years of experience) were asked to determine the IDH status of the recruited cases based on the abovementioned features. Differences of opinion were resolved by consensus for determining the final IDH status.

### 2.3 Transformed MRI textures

#### 2.3.1 Tumor segmentation

**Figure 1:**
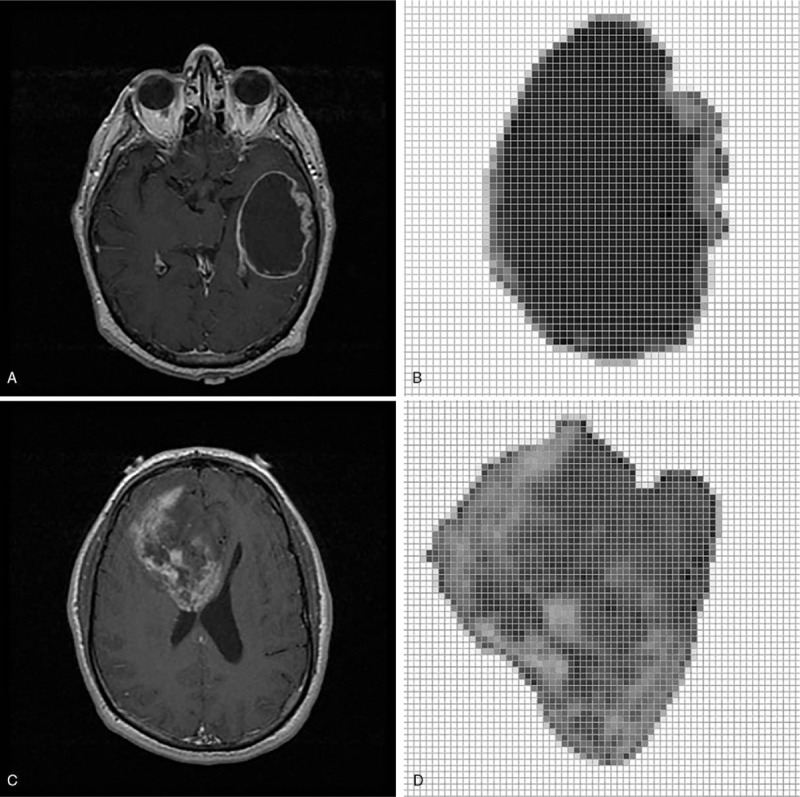
A wild-type (WT) isocitrate dehydrogenase (*IDH*) (A) and a mutant *IDH* genes (C) in magnetic resonance image. (B) and (D) are extracted tumors (http://cancerimagingarchive.net/; “License” and the CC BY license, https://creativecommons.org/licenses/by/3.0/).

Contrast-enhanced axial T1WIs were used for feature extraction for interpreting the *IDH* status. A board-certified neuroradiologist (KH), blinded to the *IDH* status information, delineated the slices with the largest axial cross-section as the representative tumor area for subsequent feature extraction for each glioblastoma. Intensity normalization, which stretched the gray-level distribution to the entire 8-bit value range (0–255) in individual images, was performed prior to contour delineation to enhance the contrast between the tumor and normal brain tissues. Contours were manually delineated with OsiriX MD (version 9.0; Pixmeo, Geneva, Switzerland). Image pixels enclosed by the delineated tumor region defined the tumor area and were used for subsequent processing and feature extraction. Figure 1A and C shows the WT *IDH* and mutant *IDH*, respectively. The corresponding tumor areas are illustrated in Figure 1B and D.

#### 2.3.2 Ranklet transformation

The image textural analysis is widely used to characterize tissues in CAD systems. Due to variations in gray-scale distributions under different scanner models and settings, textural features might not perform as well as shape features. Shape features can be extracted because the brightness between the tumor boundary and background tissues is clear. However, the contrast between pixels in a texture pattern may not strong enough. This phenomenon limits the usefulness of applying textural features in clinical diagnoses. To make the textural features more reliable, ranklet transformation was proposed to change the original pixel values into relative coefficients.^[25]^ Ranklet transformation changes the absolute gray-scale values into relative ranklet coefficients calculated by the ranked values of pixels in the local pattern to enhance the contrast.

**Figure 2:**
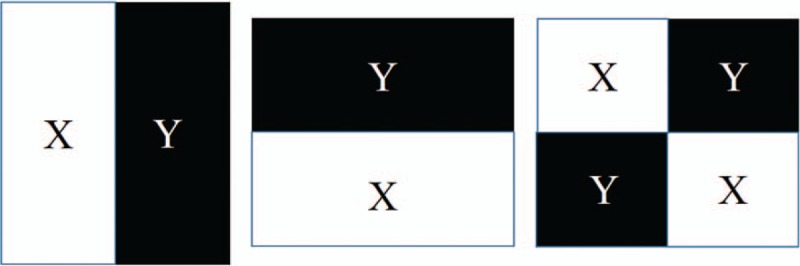
Illustration of three orientations used in the ranklet transformation, including vertical, horizontal, and diagonal patterns.

**Figure 3:**
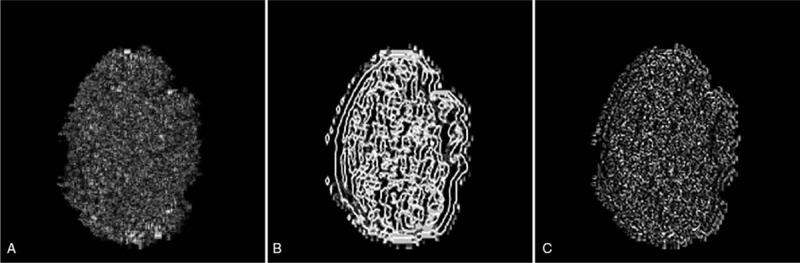
Resulting images after transforming the original tumor image in Figure 1B to a ranklet coefficient image. (A) Transformed vertical image, (B) transformed horizontal image, and (C) transformed diagonal image (http://cancerimagingarchive.net/; “License” and the CC BY license, https://creativecommons.org/licenses/by/3.0/; tumor areas in this figure were extracted from original images).

Ranklet transformation is orientation selective. If we simply separate patterns into different orientations for the textural analysis, the use of the three orientations, that is, vertical, horizontal, and diagonal, would be sufficient. The relative difference between 2 sides of a block in the vertical, horizontal, and diagonal orientations can reveal corresponding fluctuations. These blocks were separated from the original image under a resolution value (4 × 4 in the experiment). Each block was then divided into 2 subsets, *X* and *Y*, according to the selected orientations, as shown in Figure 2. Divisions, including vertical, horizontal, and diagonal orientations, were from Haar functions used in the wavelet transform^[26]^ to show local patterns. The number of pixel pairs (*P*_*H*_, *P*_*L*_) in each block was determined; that is, the relative rank of pixels of *P*_*H*_ in a subset such as *X* is higher than that of *P*_*L*_ in the other subset such as *Y*. If there are *C* pixels in a block, *C*/2 × *C*/2 = *C*^2^/4 comparisons are calculated. The resulting number was normalized to between −1 and 1. The ranklet transformation coefficient, *R*_*O*_, is formulated as follows: 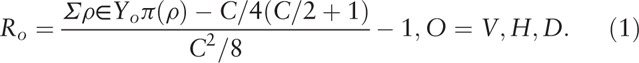


In subset *Y*_*O*_, pixel ranks *π*(*p*) are summed. If more pixels in *Y*_*O*_ are higher than those in *X*_*O*_, *R*_*O*_ is close to 1. Otherwise, it is close to −1. For patterns without strong variations, the coefficient is close to 0. By replacing the original pixel values by ranklet coefficients, the regularity correlation in the local pattern can be observed as shown in Figure 3.

#### 2.3.3 Textural features

The ranklet transformation changes the presentation of an image pattern. To extract textural features from the transformed image pattern, a computational statistical analysis is needed to quantify the pattern information to become textural features. As described in detail previously,^[27–29]^ the gray-level co-occurrence matrix (GLCM) was proposed to be promising in interpreting image textures. The GLCM texture describes the local pattern formed by correlations between adjacent pixels and is used in various CAD systems for tumor classification.^[30]^ In general, the 0 to 255 gray-scale values are reduced to generate an image, *G*, with fewer intensity bins for computational efficiency. A matrix is then established by counting the co-occurrence frequencies of two adjacent pixel values (*i* and *j*) at a distance *d* and direction *θ*.^[27]^ Settings used in this experiment were *d* = 1 and *θ* = 0°, 45°, 90°, and 135°, which were individually calculated and averaged in combination. In total, 14 GLCM textural features were implemented as below:              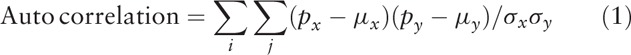



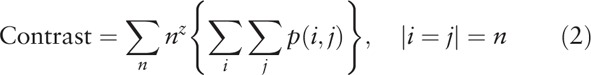



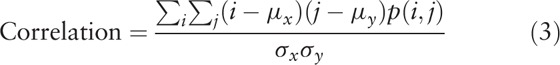



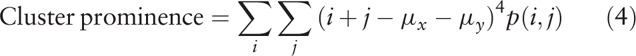



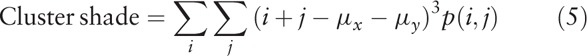



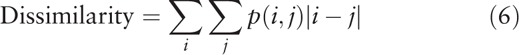



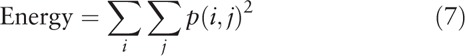



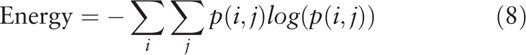



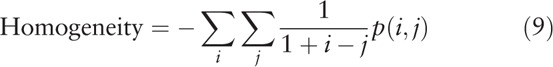



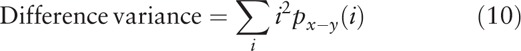



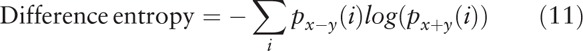



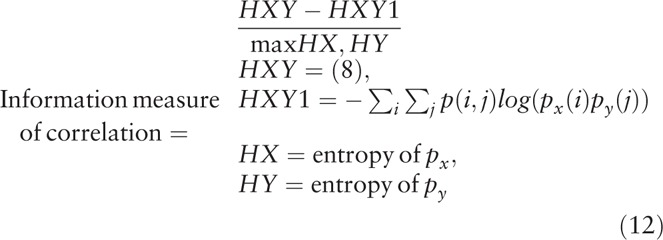



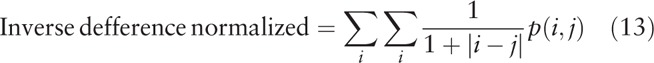



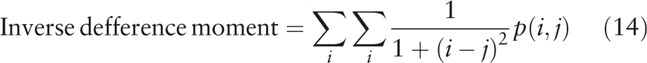


where *μ*_*x*_, *μ*_*y*_, *σ*_*x*_, and *σ*_*y*_ are the mean and standard deviation (SD) of the marginal distributions of *p*(*i,j|d,θ*).  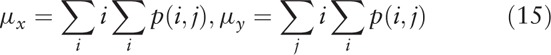



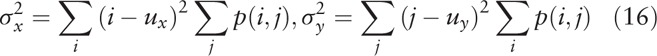


Gray-level run length matrix (GLRLM)^[31]^ was also used in the experiment for comparison. GLRLM gives the number of homogeneous runs for each gray level. The setting of GLRLM is slightly different from GLCM; GLRLM does not calculate the pair of gray scales owned, but has a length.

### 2.4 Statistical analysis

Textural features extracted from transformed MRIs, including vertical, horizontal, and diagonal feature sets, were used in the experiment to interpret the *IDH* status. Each feature set had 14 GLCM textural features describing correlations between pixels and their neighbors. With next-generation sequencing-based molecular profiles as the gold standard, features in individual categories were combined together in machine learning classifiers including logistic regression,^[32]^ k nearest neighbor (KNN),^[33]^ and support vector machine (SVM).^[34]^ Using stepwise backward elimination, the most favorable combination of features was selected with the lowest error rate. Meanwhile, the corresponding fitting model was validated using the leave-one-out method^[35]^ to determine its generalizability. While *N* is the total number of cases, an individual case was picked in each iteration and was used to validate the trained model from the other *N* – 1 cases. As a result, each case had a probability of being an *IDH* mutation according to the fitting model. Performances between different feature sets, such as the accuracy, sensitivity, specificity, positive predictive value (PPV), and negative predictive value (NPV) were compared using a Chi-squared test in SPSS (vers. 16 for Windows; SPSS, Chicago, IL, USA). The distinguishing ability of using a single feature was also tested. After evaluating whether the distribution was normal by the Kolmogorov–Smirnov test,^[35–37]^ Student *t* test^[35–37]^ was used to test features with normal distributions, and non-normal features were tested by the Mann–Whitney *U* test.^[35–37]^ A *P* value of <.05 indicated statistical significance.

## 3 Results

### 3.1 Machine learning interpretation

**Figure 4:**
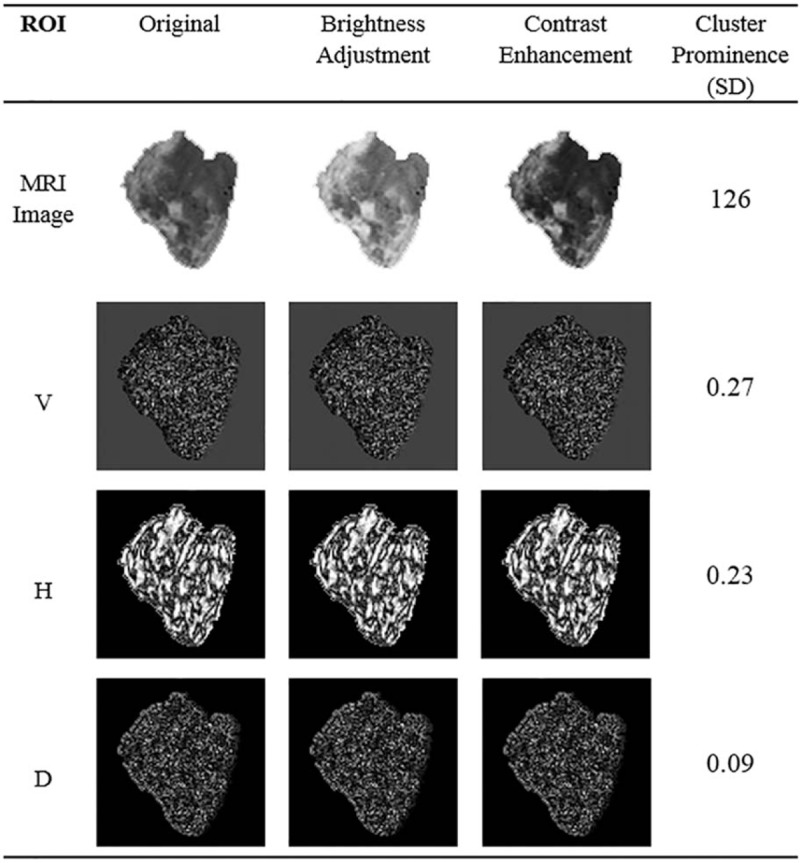
The standard deviations of feature values obtained from image processings and Ranklet transformation (http://cancerimagingarchive.net/; “License” and the CC BY license, https://creativecommons.org/licenses/by/3.0/; tumor areas in this figure were extracted from original images).

This study proposed interpreting the characteristics of glioblastomas in MRIs to predict the status of *IDH* mutations. Via ranklet transformation and GLCM textural features, 3 feature sets were extracted: vertical, horizontal, and diagonal orientations of tumor patterns. Each feature set had 14 GLCM textural features implemented (autocorrelation, contrast, correlation, cluster prominence, cluster shading, dissimilarity, energy, entropy, homogeneity, difference variance, difference entropy, information measure of correlation, inverse difference normalized, and inverse difference moment)^[27–29]^ that were combined in a logistic regression classifier to generate the prediction model. Ranklet features with the vertical orientation achieved the best performance and compared to conventional GLCM features in Table 2. Nevertheless, ranklet features obtained 90% (35/39) accuracy which is higher than GLCM features: 85% (33/39). KNN and SVM achieved accuracy of 82.1%, respectively, while the texture features of GLRLM is 85%. The GLCM features selected in the classifier included the homogeneity, difference variance, difference entropy, information measure of correlation, inverse difference normalized, and inverse difference moment. Homogeneity expresses whether tissue compositions are similar or diverse. The difference variance indicates the variance between the co-occurrence probabilities along different (*x* and *y*) axes. Correlation is the gray-scale linear dependence between a pixel and its adjacent neighbors. The inverse difference moment is also proposed to estimate the homogeneity of an image pattern.^[38]^ Taking Figure 1D as an example, the result showed a homogeneity of 0.987 and a difference variance of 0.029 which led to a 99% probability of being a mutant *IDH*. Four of them were statistically significant (*P* < .05) in distinguishing WT *IDH* and mutant *IDH*, while the other 2 features were nearly significant by Student *t* test (Table 3). Figure 4 shows that the use of ranklet transform can help to reduce the influence of varying scanning parameters and machines cross institutions on the image intensity. With respect to the feature, cluster prominence, the SD between the original image, brightness adjustment, and contrast enhancement is 126. After ranklet transformation, SD ranged from 0.09 to 0.27 for transformed original image, brightness adjustment, and contrast enhancement.

**Table 2:**
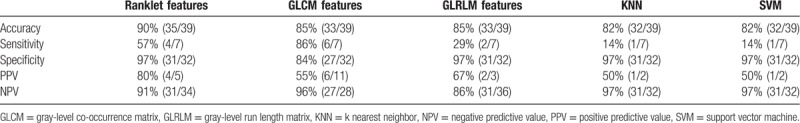
Performances of different image orientation features for predicting isocitrate dehydrogenase mutations.

**Table 3:**
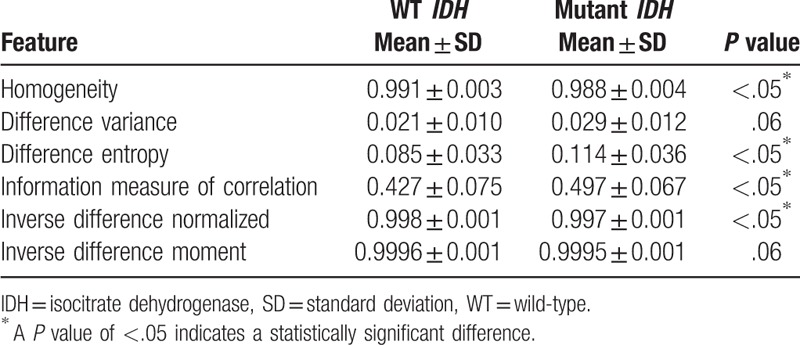
The selected textural features in the logistic regression classifier and the corresponding *P* values evaluated using Student *t* test.

### 3.2 Traditional interpretation

To determine if the proposed transformed radiomic patterns have better accuracy for the IDH mutation status than traditional interpretations, 3 neuroradiologists were asked to decide the IDH status of the recruited cases based on the MRI observation. Results showed that all five performance indices of the proposed CAD system were better than the traditional interpretation of the IDH status of GBM. The differences of accuracy and PPV were especially significant better (90% vs 72% and 80% vs 17%, respectively) as shown in Table 4. The individual performance of the 3 radiologists is listed in Table 5.

**Table 4:**
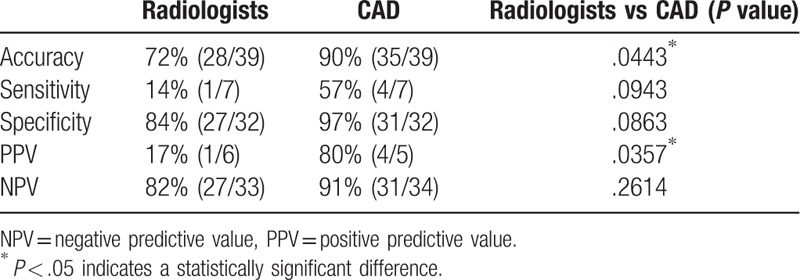
Performance comparisons between three radiologists and the proposed computer-aided diagnosis (CAD) in classifying the isocitrate dehydrogenase status.

**Table 5:**
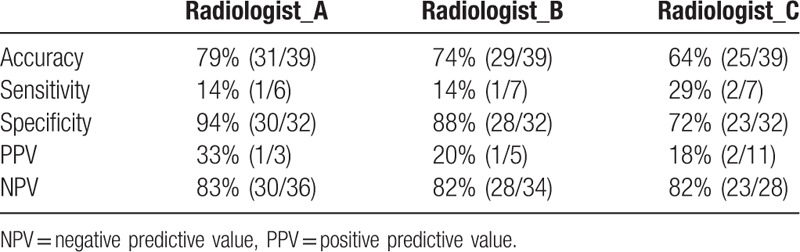
Performances of three radiologists in classifying the isocitrate dehydrogenase status.

## 4 Discussion

Several frequent mutations in *IDH* genes were unveiled by exomic sequencing,^[4,5]^ which impaired *IDH1*'s function of converting isocitrate to α-ketoglutarate and confer a gain of function in converting α-ketoglutarate to d-2HG.^[5,6]^d-2HG is thought to be an oncometabolite. It can induce epigenetic changes that result in dysregulation of gene expressions and disturbed control of cellular differentiation, leading to tumorigenesis.^[7,8]^ IDH mutations are highly selective molecular biomarkers of secondary disease because these mutations are mainly observed in secondary GBMs.^[8,9]^ Therefore, tumors with mutant *IDH* genes are believed to have a more heterogeneous compositions and imaging characteristics because of the stepwise gliomagenesis pattern of secondary GBMs.^[17]^

According to our previous study,^[39]^ textural features describing heterogeneous patterns were extracted from MRIs and combined in the classifier to correctly classify 33 of 39 *IDH* mutation types. The difference in accuracy between our previous study^[39]^ and this study was not significant. Nevertheless, this study further achieved 90% accuracy which is higher than previous 85%. The proposed ranklet-transformed features achieved less *P* values or very close to .05 which were better than our previous results using pure textural features (Table 3). Several classifiers were tried including KNN, and support vector machine with or without principal components analysis to explore any better feature combinations. The resulting highest accuracy is 82.1% which is no better than the proposed method. Other texture features such as GLRLM was also used in the experiment. With ranklet transformation or without, GLRLM only achieved the best accuracy of 85%. The matrix compositions are different from GLCM. The use of ranklet may be only suitable for matrix form of GLCM.

Image intensity variance caused by different scanning parameters and machines may have an influence on the diagnosis. Ranklet-transformed textural features emphasize local contrasts using relative coefficients that may better present specific heterogeneous patterns. This technique was 1st applied to reduce the effects of different scanning parameters and machines on the underlying patterns. As shown in Figure 4, the SDs between various gray-scale compositions such as the variations of brightness and contrast were eliminated after ranklet transformation. Additionally, our results depicted that tumors with *IDH* mutations had lower homogeneity. The combination of these imaging characteristics suggested that mutant *IDH* GBMs tended to have more-heterogeneous imaging intensities, which also implied their multistage tumorigenic behaviors.

Using a radiomic model for predicting *IDH* mutations provides a connection between intuitive vision and precision medicine. Tumor characteristics can be mapped and quantified by applying high throughput radiomic analysis on routine MRI examination without requiring a risky surgery. However, this preliminary study was limited by the insubstantial number of mutant *IDH* cases. More cases should be collected in further studies to support the above results. However, we did our best to enroll cases from 4 hospitals and maintained a ratio between WT *IDH* and mutant *IDH* to provide these preliminary results. Another limitation is that only contrast-enhanced T1WIs were used in our analysis, which are insufficient to characterize peritumoral edema. Nevertheless, *IDH* mutations are linked to angiogenesis,^[9]^ and it was reported that the activity of the angiogenesis module in a tumor was associated with the signal intensity of contrast enhancement.^[40,41]^ Therefore, it is reasonable that the imaging features extracted from contrast-enhanced T1WIs can be applied to predict whether GBMs have *IDH* mutations. The impact of other MRI sequences including apparent diffusion coefficient map, perfusion-weighted imaging, and diffusion tensor imaging will be further investigated.

## 5 Conclusion

A CAD system was proposed to interpret the status of *IDH* in glioblastomas from transformed MRI patterns. Quantitative textural features extracted from the transformed ranklet images achieved an accuracy of 90%, a sensitivity of 57%, and a specificity of 97%. The system based on textural features from ranklet-transformed images is a promising noninvasive method to provide suggestions about the *IDH* status in GBM.

## Acknowledgments

The authors thank the Ministry of Science and Technology, Taiwan (MOST104-2218-E-038-004 and 105-2314-B-038-049), and Taipei Medical University and Taipei Medical University Hospital (106TMU-TMUH-20) for financially supporting this study.

## Author contributions

**Conceptualization:** Kevin Li-Chun Hsieh.

**Data curation:** Sho-Jen Cheng, Hung-Jung Wang.

**Funding acquisition:** Rui-Cian Weng.

**Methodology:** Chung-Ming Lo.

**Resources:** Rui-Cian Weng.

**Software:** Chung-Ming Lo.

**Validation:** Sho-Jen Cheng, Hung-Jung Wang.

**Writing – original draft:** Chung-Ming Lo, Kevin Li-Chun Hsieh.

**Writing – review & editing:** Kevin Li-Chun Hsieh.

## References

[1] Wen PY Kesari S Malignant gliomas in adults. N Engl J Med 2008;359:492–507. 18669428 10.1056/NEJMra0708126

[2] Louis DN Ohgaki H Wiestler OD The 2007 WHO classification of tumours of the central nervous system. Acta Neuropathol 2007;114:97–109. 17618441 10.1007/s00401-007-0243-4 PMC1929165

[3] Louis DN Perry A Reifenberger G The 2016 World Health Organization Classification of Tumors of the Central Nervous System: a summary. Acta Neuropathol 2016;131:803–20. 27157931 10.1007/s00401-016-1545-1

[4] Parsons DW Jones S Zhang X An integrated genomic analysis of human glioblastoma multiforme. Science 2008;321:1807–12. 18772396 10.1126/science.1164382 PMC2820389

[5] Yan H Parsons DW Jin G IDH1 and IDH2 mutations in gliomas. N Engl J Med 2009;360:765–73. 19228619 10.1056/NEJMoa0808710 PMC2820383

[6] Dang L White DW Gross S Cancer-associated IDH1 mutations produce 2-hydroxyglutarate. Nature 2009;462:739–44. 19935646 10.1038/nature08617 PMC2818760

[7] Lu C Ward PS Kapoor GS IDH mutation impairs histone demethylation and results in a block to cell differentiation. Nature 2012;483:474–8. 22343901 10.1038/nature10860 PMC3478770

[8] Turcan S Rohle D Goenka A IDH1 mutation is sufficient to establish the glioma hypermethylator phenotype. Nature 2012;483:479–83. 22343889 10.1038/nature10866 PMC3351699

[9] Pelloski CE Ballman KV Furth AF Epidermal growth factor receptor variant III status defines clinically distinct subtypes of glioblastoma. J Clin Oncol 2007;25:2288–94. 17538175 10.1200/JCO.2006.08.0705

[10] Weller M Felsberg J Hartmann C Molecular predictors of progression-free and overall survival in patients with newly diagnosed glioblastoma: a prospective translational study of the German Glioma Network. J Clin Oncol 2009;27:5743–50. 19805672 10.1200/JCO.2009.23.0805

[11] Cryan JB Haidar S Ramkissoon LA Clinical multiplexed exome sequencing distinguishes adult oligodendroglial neoplasms from astrocytic and mixed lineage gliomas. Oncotarget 2014;5:8083–92. 25257301 10.18632/oncotarget.2342 PMC4226668

[12] Beiko J Suki D Hess KR IDH1 mutant malignant astrocytomas are more amenable to surgical resection and have a survival benefit associated with maximal surgical resection. Neuro Oncol 2014;16:81–91. 24305719 10.1093/neuonc/not159 PMC3870823

[13] Jackson A O’Connor JP Parker GJ Imaging tumor vascular heterogeneity and angiogenesis using dynamic contrast-enhanced magnetic resonance imaging. Clin Cancer Res 2007;13:3449–59. 17575207 10.1158/1078-0432.CCR-07-0238

[14] Jain R Poisson L Narang J Genomic mapping and survival prediction in glioblastoma: molecular subclassification strengthened by hemodynamic imaging biomarkers. Radiology 2013;267:212–20. 23238158 10.1148/radiol.12120846 PMC3606543

[15] Andronesi OC Kim GS Gerstner E Detection of 2-hydroxyglutarate in IDH-mutated glioma patients by in vivo spectral-editing and 2D correlation magnetic resonance spectroscopy. Sci Transl Med 2012;4:116ra4. 10.1126/scitranslmed.3002693 PMC3720836 22238332

[16] Choi C Ganji SK DeBerardinis RJ 2-hydroxyglutarate detection by magnetic resonance spectroscopy in IDH-mutated patients with gliomas. Nat Med 2012;18:624–9. 22281806 10.1038/nm.2682 PMC3615719

[17] Lee S Choi SH Ryoo I Evaluation of the microenvironmental heterogeneity in high-grade gliomas with IDH1/2 gene mutation using histogram analysis of diffusion-weighted imaging and dynamic-susceptibility contrast perfusion imaging. J Neurooncol 2015;121:141–50. 25205290 10.1007/s11060-014-1614-z

[18] Chang R-F Lee C-C Lo C-M Computer-aided diagnosis of different rotator cuff lesions using shoulder musculoskeletal ultrasound. Ultrasound Med Biol 2016;42:2315–22. 27381057 10.1016/j.ultrasmedbio.2016.05.016

[19] Hsieh KL-C Lo C-M Hsiao C-J Computer-aided grading of gliomas based on local and global MRI features. Comput Methods Programs Biomed 2017;139:31–8. 28187893 10.1016/j.cmpb.2016.10.021

[20] Buch K Li B Qureshi MM Quantitative assessment of variation in CT parameters on texture features: pilot study using a nonanatomic phantom. AJNR Am J Neuroradiol 2017;38:981–5. 28341714 10.3174/ajnr.A5139 PMC7960393

[21] McLendon R Friedman A Bigner D Comprehensive genomic characterization defines human glioblastoma genes and core pathways. Nature 2008;455:1061–8. 18772890 10.1038/nature07385 PMC2671642

[22] Ellingson BM Lai A Harris RJ Probabilistic radiographic atlas of glioblastoma phenotypes. AJNR Am J Neuroradiol 2013;34:533–40. 22997168 10.3174/ajnr.A3253 PMC7964888

[23] Lai A Kharbanda S Pope WB Evidence for sequenced molecular evolution of IDH1 mutant glioblastoma from a distinct cell of origin. J Clin Oncol 2011;29:4482–90. 22025148 10.1200/JCO.2010.33.8715 PMC3236649

[24] Ellingson BM Radiogenomics and imaging phenotypes in glioblastoma: novel observations and correlation with molecular characteristics. Curr Neurol Neurosci Rep 2015;15:506. 25410316 10.1007/s11910-014-0506-0

[25] Lo C-M Moon WK Huang C-S Intensity-invariant texture analysis for classification of bi-rads category 3 breast masses. Ultrasound Med Biol 2015;41:2039–48. 25843514 10.1016/j.ultrasmedbio.2015.03.003

[26] Mallat SG A theory for multiresolution signal decomposition: the wavelet representation. IEEE 1989;11:674–93.

[27] Haralick RM Shanmugam K Dinstein IH Textural features for image classification. IEEE 1973;SMC-3:610–21.

[28] Clausi DA An analysis of co-occurrence texture statistics as a function of grey level quantization. Canad J Remote Sens 2002;28:45–62.

[29] Soh L-K Tsatsoulis C Texture analysis of SAR sea ice imagery using gray level co-occurrence matrices. IEEE 1999;37:780–95.

[30] Lo C-M Chen R-T Chang Y-C Multi-dimensional tumor detection in automated whole breast ultrasound using topographic watershed. IEEE Trans Med Imaging 2014;33:1503–11. 24718570 10.1109/TMI.2014.2315206

[31] Mohanty AK Beberta S Lenka SK Classifying benign and malignant mass using GLCM and GLRLM based texture features from mammogram. Int J Eng Res Appl 2011;1:687–93.

[32] Menard S Applied Logistic Regression Analysis. Vol. 106. US: Sage; 2002.

[33] Peterson LE K-nearest neighbor. Scholarpedia 2009;4:1883.

[34] Suykens JA Vandewalle J Least squares support vector machine classifiers. Neural Proces Lett 1999;9:293–300.

[35] Field AP Discovering Statistics Using SPSS. 3rd ed Los Angeles, CA: SAGE Publications; 2009.

[36] Hsieh KL-C Chen C-Y Lo C-M Quantitative glioma grading using transformed gray-scale invariant textures of MRI. Comp Biol Med 2017;83:102–8. 10.1016/j.compbiomed.2017.02.012 28254615

[37] Moon WK Chen I-L Chang JM The adaptive computer-aided diagnosis system based on tumor sizes for the classification of breast tumors detected at screening ultrasound. Ultrasonics 2017;76:70–7. 28086107 10.1016/j.ultras.2016.12.017

[38] Sharma N Ray AK Sharma S Segmentation and classification of medical images using texture-primitive features: Application of BAM-type artificial neural network. J Med Phys 2008;33:119–26. 19893702 10.4103/0971-6203.42763 PMC2772042

[39] Hsieh KL Chen CY Lo CM Radiomic model for predicting mutations in the isocitrate dehydrogenase gene in glioblastomas. Oncotarget 2017;8:45888–97. 28526813 10.18632/oncotarget.17585 PMC5542235

[40] Diehn M Nardini C Wang DS Identification of noninvasive imaging surrogates for brain tumor gene-expression modules. Proc Natl Acad Sci U S A 2008;105:5213–8. 18362333 10.1073/pnas.0801279105 PMC2278224

[41] Pope WB Chen JH Dong J Relationship between gene expression and enhancement in glioblastoma multiforme: exploratory DNA microarray analysis 1. Radiology 2008;249:268–77. 18796682 10.1148/radiol.2491072000 PMC2798090

